# Regenerative peripheral nerve interfaces for real-time, proportional control of a Neuroprosthetic hand

**DOI:** 10.1186/s12984-018-0452-1

**Published:** 2018-11-20

**Authors:** Christopher M. Frost, Daniel C. Ursu, Shane M. Flattery, Andrej Nedic, Cheryl A. Hassett, Jana D. Moon, Patrick J. Buchanan, R. Brent Gillespie, Theodore A. Kung, Stephen W. P. Kemp, Paul S. Cederna, Melanie G. Urbanchek

**Affiliations:** 1 University of Michigan Department of Surgery, Section of Plastic Surgery, 570 MSRB II Level A, 1150 W. Medical Center Drive, Ann Arbor, MI 48109-5456 USA; 2 University of Michigan Department of Mechanical Engineering, Ann Arbor, MI USA; 3 Vassar College, Poughkeepsie, NY USA; 4 Department of Biomedical Engineering, University of Michigan, Ann Arbor, MI USA

**Keywords:** Peripheral nerve Interface, Prosthetics, Regenerative medicine, Amputees

## Abstract

**Introduction:**

Regenerative peripheral nerve interfaces (RPNIs) are biological constructs which amplify neural signals and have shown long-term stability in rat models. Real-time control of a neuroprosthesis in rat models has not yet been demonstrated. The purpose of this study was to: a) design and validate a system for translating electromyography (EMG) signals from an RPNI in a rat model into real-time control of a neuroprosthetic hand, and; b) use the system to demonstrate RPNI proportional neuroprosthesis control.

**Methods:**

Animals were randomly assigned to three experimental groups: (1) Control; (2) Denervated, and; (3) RPNI. In the RPNI group, the extensor digitorum longus (EDL) muscle was dissected free, denervated, transferred to the lateral thigh and neurotized with the residual end of the transected common peroneal nerve. Rats received tactile stimuli to the hind-limb via monofilaments, and electrodes were used to record EMG. Signals were filtered, rectified and integrated using a moving sample window. Processed EMG signals (iEMG) from RPNIs were validated against Control and Denervated group outputs.

**Results:**

Voluntary reflexive rat movements produced signaling that activated the prosthesis in both the Control and RPNI groups, but produced no activation in the Denervated group. Signal-to-Noise ratio between hind-limb movement and resting iEMG was 3.55 for Controls and 3.81 for RPNIs. Both Control and RPNI groups exhibited a logarithmic iEMG increase with increased monofilament pressure, allowing graded prosthetic hand speed control (R^2^ = 0.758 and R^2^ = 0.802, respectively).

**Conclusion:**

EMG signals were successfully acquired from RPNIs and translated into real-time neuroprosthetic control. Signal contamination from muscles adjacent to the RPNI was minimal. RPNI constructs provided reliable proportional prosthetic hand control.

**Electronic supplementary material:**

The online version of this article (10.1186/s12984-018-0452-1) contains supplementary material, which is available to authorized users.

## Introduction

Approximately 185,000 individuals suffer limb loss annually in the United States [1]. The growing rate of amputees and technological advancements have greatly improved human-neuroprosthetic interfacing [2]. A comprehensive literature review on the needs and priorities of prostheses users performed by Cordella et al. in 2016 revealed that an estimated 75% of upper prosthetic users wore functional prostheses for at least 8 h per day, compared with only 45% of cosmetic prosthesis owners [3]. A functional prosthesis was more likely to be worn the higher the level of amputation, and especially during dynamic activities of daily living, such as work, driving and sports [3]. Importantly, upper arm amputees who tested both conventional (body powered or myoelectric arms) and the DEKA Gen 3 advanced myoelectric prosthesis found conventional prostheses performed faster, and with smoother motions and less movement deviation than the advanced DEKA prosthetic device [4]. This finding is largely attributed to a lack of an intuitive, functional neural interface that can provide high fidelity control signals to actualize the functionality of advanced neuroprosthetic devices.

Advanced anthropomorphic modular prosthetic arm systems have only become commercially available in the last 5 years, in large part due to technology developed with DARPA’s funding of the Revolutionizing Prosthetics Program in 2006 [5]. Currently, multi-electrode-based prosthetic devices, such as the DEKA arm (DEKA, Manchester, NH), i-Limb (TouchBionics, Touch Bionics, Mansfield, MA), the Johns Hopkins Modular Prosthetic Limb (MPL, Johns Hopkins University Applied Physics Lab, Baltimore, MD), and Ottobock (Otto Bock HealthCare, Duderstadt, Germany), provide increased ranges of motion, dexterity and control options, and are capable of up to five-finger movements and 20 degrees of freedom [6, 7]. However, a limitation in controlling these advanced robotic prostheses is the need for an appropriate neural interface that can extract clear multifunctional signal information at a speed that matches naturalistic human motion [8, 9].

Neural interfaces, i.e. the use of electrodes to record physiological signals for voluntary prosthetic control, come in different forms and all have unique advantages and challenges. All prostheses require either nerve or muscle electrodes as part of the neural interface [6], and consequently, interfacing electrodes vary in size (standard pad to microelectrodes), shape (multipolar cuff, fine wire, sieve), number of electrode sites (bipolar or multi-array), and location (transverse intrafascicular multichannel nerve, longitudinal intrafascicular nerve, epimysial, intramysial and intracortical microelectrode arrays placed in the cortex) [9–12]. Cuff electrodes circumferentially envelope peripheral nerves and nerve fascicles, and have shown promising results in signal transduction; however, long term signal fidelity may be compromised due to epineurial inflammation and scarring [13–15]. Both intrafascicular electrodes and sieve electrodes allow for nerve and signal specificity, but are hampered by long-term signal loss due to biofouling [16–18]. Epimysial and intramysial electrodes can be larger in size, are physically more robust, are less compromised by fibrosis, and transduce myoelectric signals with less impedance [19].

The most successful form of neural interfacing to date is Targeted Muscle Reinnervation (TMR) [20]. TMR is an FDA-approved procedure to surgically construct additional EMG control sites using residual nerves [21]. Remaining nerves from the amputated limb are transferred to expendable regions of residual muscle in or near the residual limb; commonly, the ipsilateral pectoral muscle is denervated and used for this purpose. The nerves reinnervate the “target” and produce additional EMG signal sites for prosthetic control. Ideally, TMR is performed during the initial amputation procedure, which has been proven to reduce neuroma formation [22–24]. TMR uses external skin surface electrodes to transduce EMG signals, thus avoiding the build-up of connective tissue on electrodes due to a foreign body reaction. Yet a disadvantage of surface EMG electrode systems is their lack of robustness to variance caused by donning, fatigue, perspiration, and other conditions that cause positional and physiological changes in the electrical characteristics of the signal sites [21]. Moreover, the reinnervation of the whole pectoral muscle with up to three nerves, each of which is responsible for specific and distinct functions in the arm, requires the implementation of complex pattern classification and feature extraction algorithms, such that the overlapping neural signals acquired from the EMG electrode array can be decoded and assigned to their intended control targets [25].

Despite the advancements that have benefitted human-prosthetic interfacing, a need remains for a neural interface that can provide real-time, long-term, contamination free, signal fidelity for optimal prosthetic activation and control. In this study, we use the Regenerative Peripheral Nerve Interface (RPNI) as a strategy for neural interfacing. RPNIs are neuromuscular biological interfaces surgically constructed from free muscle grafts (3 × 1 cm.) obtained from expendable skeletal muscle in the residual limb or from a distant site. The residual peripheral nerves are dissected into single nerve fascicles, or groups of fascicles, to create functional units. The muscle grafts are then neurotized by the terminal branches of the residual nerves. Revascularization, regeneration, and eventually reinnervation allows the RPNI to mature in 3 to 4 months [26, 27]. This technique reduces the amount of neural manipulation and risk of iatrogenic nerve damage. Previous studies in our laboratory have shown that RPNIs transduce evoked muscle potentials for up to 18 months, prevent neuroma formation, and amplify motor nerve signaling [28, 29]. Thus, RPNI technology takes advantage of the signal from individual muscles that can be recorded via intramuscular EMG signals generated from the RPNI, obviating the need for signal decoding of multi-nerve motor features via classification algorithms [21].

There have been few investigations into the fine motor control of neuroprosthetic devices using the RPNI technique. As such, the purposes of this study were to: a) build and validate an algorithm for translating EMG signals from RPNIs for real-time control of a myoelectrically actuated neuroprosthetic hand; and b) use this algorithm to demonstrate the ability of RPNIs to provide proportional neuroprosthesis control. It was hypothesized that both Control and RPNI groups would demonstrate reliable and proportional control of the myoelectric hand, while the Denervated group would not activate the neuroprosthesis.

## Methods

### Animal model

All procedures were approved by the University of Michigan, Institutional Animal Care and Use Committee, and were in strict accordance with the National Research Council’s *Guide for the Care and Use of Laboratory Animals* (1996) [30]. Retired F344 male breeder rats (Charles River, Wilmington, MA) weighing 300 to 420 g were anesthetized with weight-based Pentobarbital and administered Buprenorphine-HCl as analgesia.

### Regenerative peripheral nerve Interface surgery

**Fig. 1:**
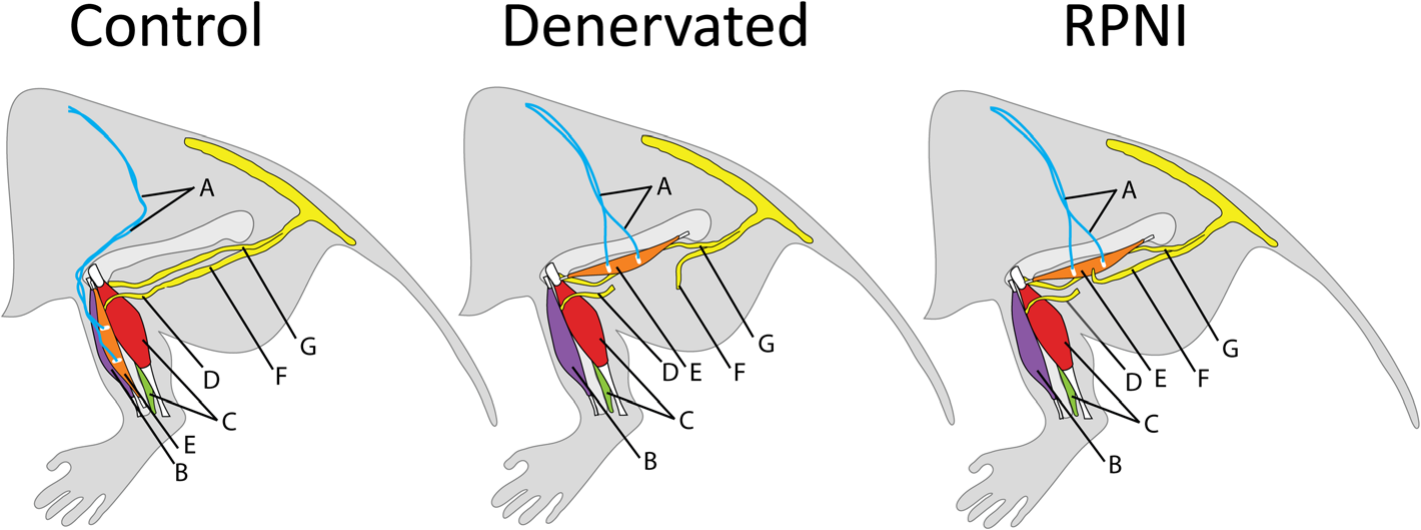
Left: Control group with primary repair of the extensor digitorum longus muscle (EDL) tenotomies without denervation of the muscle. Center: Denervated group with free EDL muscle graft performed to the lateral thigh. Neurotization and reinnervation was not performed, leaving the EDL muscle graft without innervation. Electrode placement was identical to the Control group. Right: Regenerative Peripheral Nerve Interface (RPNI) group with free EDL muscle graft performed to the lateral thigh. Neurotization and reinnervation were implemented using the peroneal nerve. Each rat received bipolar epimysial electrodes (white), whose wires (blue) were tunneled subcutaneously to the upper dorsum. **a.** bipolar electrode cables. ***b.*** *tibialis* anterior muscle; **c.** soleus and gastrocnemius muscles; **d.** distal end of common peroneal nerve; **e.** EDL muscle; **f.** proximal common peroneal nerve; **g.** tibial nerve

The study design consisted of three separate groups, Control (*n* = 2), Denervated (*n* = 1), and RPNI (*n* = 3). In each group, all rats underwent a proximal and distal tenotomy of the extensor digitorum longus (EDL) muscle. In the Control group, no additional interventions were performed. In the Denervated and RPNI groups, the common peroneal nerve was divided and the free EDL muscle graft was transferred to the lateral thigh. In the RPNI group, the proximal end of the divided peroneal nerve was implanted into the EDL skeletal muscle graft to create an RPNI. In the Denervated group, the proximal end of the peroneal nerve was reflected proximally to prevent EDL skeletal muscle graft reinnervation (Fig. 1).

Two stainless steel electrodes made of Cooner wire (Cooner Wire Co., Chatsworth, CA) were sutured onto the EDL epimysium, with electrodes separated longitudinally by 1.5 cm. The EDL muscle was then covered by a single-layer of acellular porcine intestinal submucosa scaffold (SIS) (Surgisis, Cook Biotech, West Lafayette, IN). The leading ends and connecting cables of the electrodes were tunneled, coiled, and buried subcutaneously within the dorsum of each rat between the scapulae.

### Testing protocol

Five months following implantation, the free ends of the implanted electrode cables were exposed through a dorsal incision. EMG signals were then recorded, amplified to 1000x and band-pass filtered (1–500 Hz) on a custom-built analog bipolar instrumentation amplifier. Signal amplitudes were calibrated using a function generator (B&K Precision, Model 4075, B&K Precision Corporation, Yorba Linda, CA) and oscilloscope (Agilent InfiniiVision Model MSO-X 2012-A, Agilent Technologies, Santa Clara, CA). The amplified and filtered signals were acquired at a 3 kHz sampling rate using a data acquisition card (NI BNC 2120, National Instruments, Austin, TX) using LabVIEW software (National Instruments, Austin, TX). During post processing, the signals were digitally rectified and zero-phase low-pass filtered to 50 Hz.

**Fig. 2:**
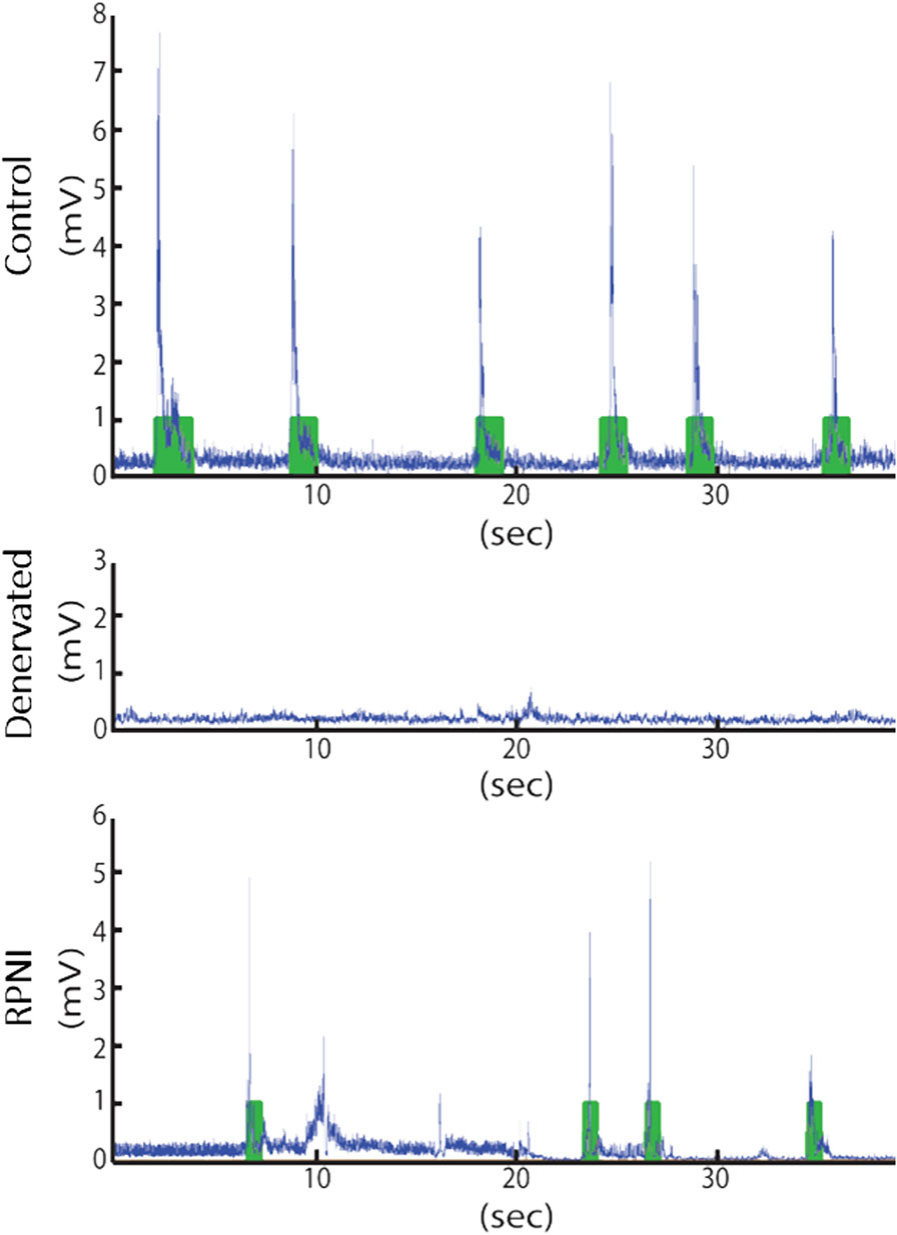
EMG signals integrated over 300 msec (iEMG) – based prosthesis activation during one testing session. Plots of filtered EMG tracings (Blue) and periods of prosthesis activation (Green) during 40 s of testing in Control (Top), Denervated (Middle) and RPNI (Bottom) groups. Baseline iEMG is calculated as a running average. An algorithm activates the prosthesis after detecting an iEMG window more than 1 standard deviation above the mean iEMG

A von Frey monofilament testing protocol was initiated to evoke reflex anterior compartment dorsi-flexion of the hind paw, and activation of the EDL or RPNI muscle [31]. During testing, each rat was placed in a 4 × 5 × 8 in.^3^ Plexiglas® box with a wire mesh bottom. Monofilament fibers were applied to the left experimental ankle to induce a voluntary muscle reflex leg movement. Monofilament pressure was initiated at 4 g of force, and monofilament fibers of up to 100 g were randomly administered to the ankle. Four cycles lasting five minutes were performed at each monofilament force level. All rats were free to ambulate while connected to the myoelectric prosthesis to correct for the possibility of EMG signaling from other muscles. To avoid habituation, 1–2 min of rest was allowed between each testing cycle. Rats in each group were evaluated for 3 days with 2 days of rest between each evaluation period. The monofilament testing lasted no longer than 2 h per day. Post-evaluation, all rats were sacrificed and their hind limb dissected in order to assess the amount of scar tissue and vascularity in the repaired EDL (Control group) and free grafted muscles in the lateral thigh (RPNI and Denervated groups). Prosthetic activation and hind limb movement were video recorded at 120 frames per second using a high-speed, high-definition camera (GoPro Hero2, San Mateo, CA). Rectified EMG and prosthetic activation were synchronized and recorded using the LabVIEW software (Fig. 2).

### Algorithm design

**Fig. 3:**
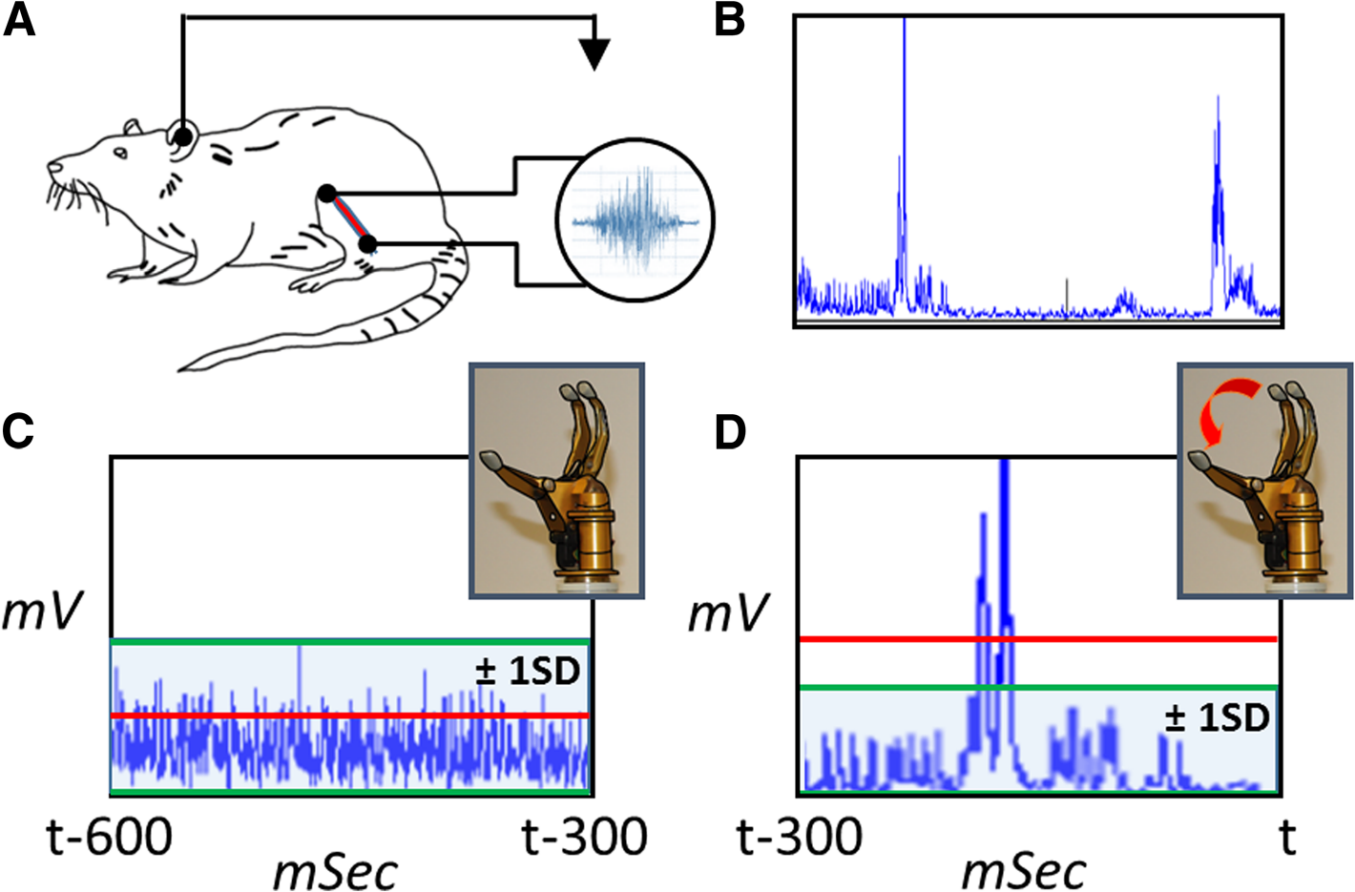
Schematic showing acquisition, transduction and analysis of real-time recorded EMG signaling from an RPNI rat. **a.** Bipolar collection of raw EMG signals. Ground electrode is referenced in ear. **b.** Raw EMG signals undergo signal processing in the form of filtering and rectification. **c. & d.** 300 msec consecutive EMG signal acquisition intervals obtained during **c.** no observed leg motion (baseline signal activity below threshold), and **d.** Leg motion and subsequent prosthetic hand activation due to signal surpassing threshold of activation. Blue lines: EMG signal; Red lines: iEMG value; Green lines: Activation threshold

A computer algorithm was written using LabVIEW to allow interpretation of the EMG activity and prosthetic control. The rectified and filtered EMG signals were divided into 300 millisecond intervals. Each 300 millisecond interval was then integrated with respect to time and a mean value iEMG was calculated in units of mV × sec. A running threshold was calculated by averaging all previous intervals, giving 50% weight to the immediately prior interval. Activation of the prosthesis occurred when the real-time iEMG was greater than the running threshold by at least one standard deviation (Fig. 3).

Graded control of the prosthesis was achieved by modulation of the output voltage to the “DMC + Hand” (Otto Bock Healthcare, Vienna, Austria) using an Arduino Uno R3 prototyping board (Arduino LLC. Cambridge, MA) equipped with a motor-driving amplifier (SparkFun Electronics, Niwot, CO). Output voltage to the hand was increased with larger iEMG values by calculating the number of standard deviations above the running threshold for each iEMG interval (Eq. 1).

1 $$ {V}_{O\mathrm{u} tput}={V}_{Max}-\frac{V_{Max}}{1+\left({SD}_{Above\ Threshold}\right)} $$

### Data analysis

Video recordings of each testing period were analyzed to determine interface performance. Sensitivity and specificity were each calculated based on appropriate activation of the prosthesis during hind limb movement and non-activation during periods of rest, respectively. The number of recorded prosthetic movements during each 4-min testing period was compared to the total number of observed leg movements to determine sensitivity (Eq. 2). The number of errant activations of the prosthesis during rest intervals with no hind limb movement was calculated to determine specificity (Eq. 3). Within group Student’s T-Test statistical computations were performed using SPSS Statistics 22, (SPSS, IBM Inc., 2013, Armonk, NY). Significance levels were set to α = 0.05.

2 $$ Sensitivity=\frac{Prosthetic\ activations\ after\ hindlimb\ movement\ }{Total\ number\ of\ hindlimb\ movement s} $$

3 $$ Specifity=\frac{Total\ rest\ intervals- prosthetic\ activations\ during\ rest\ intervals\ }{Total\ rest\ intervals} $$

## Results

### Accuracy of Neuroprosthesis activation

In total, 1040 Control group hind limb movements in 208 min and 876 RPNI group hind limb movements in 172 min were captured. (see Video, Additional file 1: Video S1, which demonstrates prosthesis activation in response to monofilament stimulation on the volar side of the hind paw) Significantly reduced hind paw movements were recorded during 51 min within the Denervated group, likely resulting from the lack of peroneal nerve innervation to the lateral compartment musculature of the lower hind limb (see Video, Additional file 2: Video S2, which demonstrates no prosthesis activation in response to monofilament stimulation on the volar side of the hind paw in a Denervated rat).


Additional file 1: **Video S1.** Which illustrates prosthetic hand activation using RPNI generated myoelectric signals, in response to monofilament application to the left hind paw of a rat fitted with an RPNI. (MP4 12600 kb)
Additional file 2: **Video S2.** Which illustrates a denervated rat experimental trial, in which monofilament application fails to produce actuation of the prosthetic hand. (MP4 4092 kb)


The iEMG activation signals were significantly higher in both the Control and RPNI groups when compared to the baseline signals obtained during the between-trial resting periods, indicating that the calculated threshold denoting prosthesis activation (Eq. 1) was successfully defined. The calculated sensitivity (ability to detect prosthetic activation after stimulation) and specificity (ability to prevent unwanted activation during rest) values for prosthesis activation are reported in Table 1. Signal to noise ratio means and standard deviations between iEMG resulting in initial hind limb movement, (i.e. iEMG acquired during the lowest monofilament stimulus resulting in paw retraction, and therefore prosthesis activation) and resting iEMG was 3.55 ± 0.38 and 3.81 ± 0.52 for the Control and RPNI groups, respectively.

**Table 1:** Summary Data of EMG Translation System

Dependent variables	SURGICAL GROUPS		
	Control (n = 2 rats)	Denervated (n = 1 rat)	RPNI (n = 3 rats)
Mass (g) on test day	420	397	302
Sensitivity	0.902 (0.06)	^a^	0.879 (0.08)
Specificity	0.998 (0.004)	1.0 (0.0)	0.988 (0.02)

Values are means (± 1 SD). Sensitivity and specificity were excellent across all three groups. ^a^ Denervated group as expected did not show activity during rat movement; therefore no sensitivity was calculated

### Proportional control of the Neuroprosthesis

**Fig. 4:**
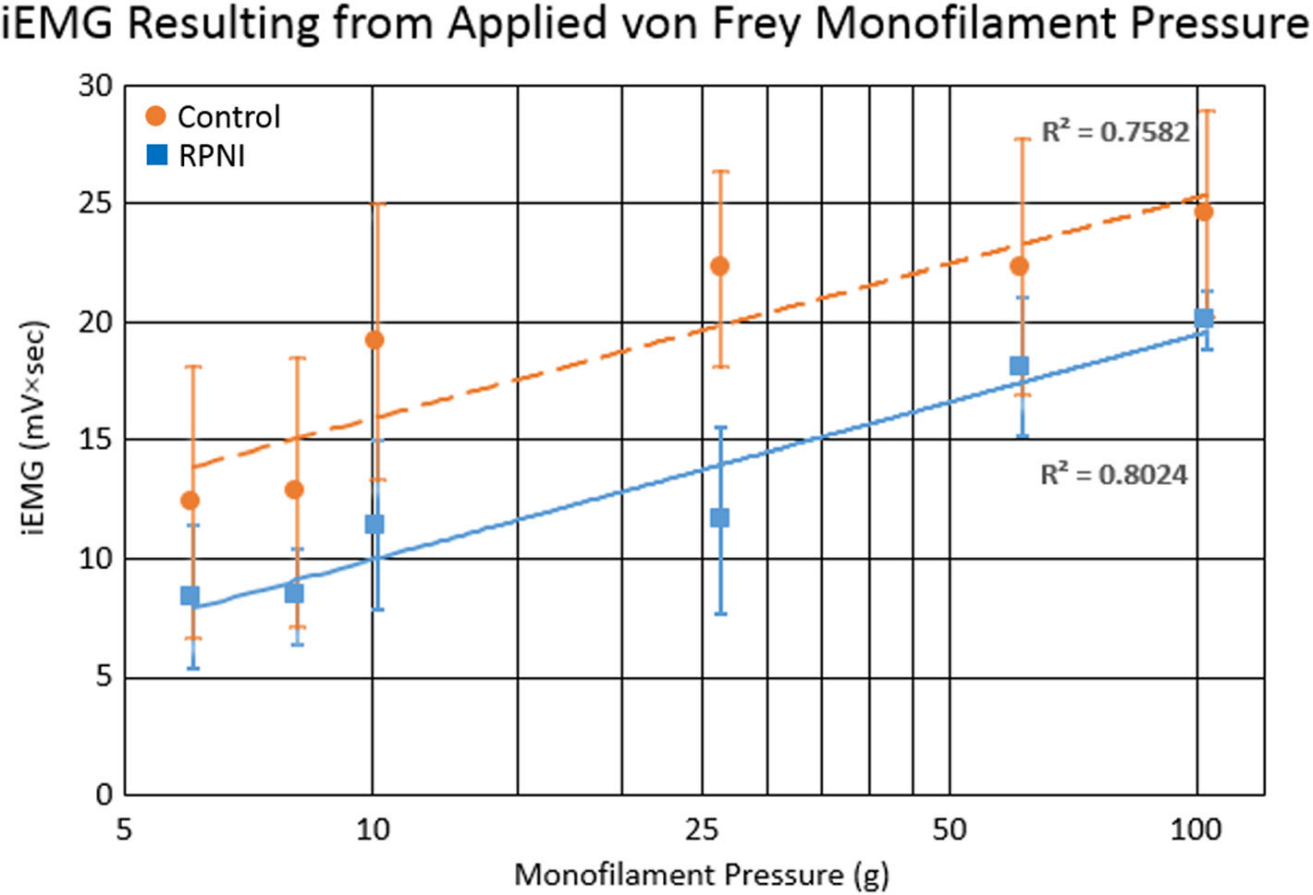
A semi-logarithmic relationship between monofilament pressure applied and iEMG recorded during four testing blocks. Each block lasted 5 min for each increment of pressure increase in RPNI and Control groups (blue and orange, respectively). Monofilament pressure is graphed logarithmically to linearize each graph. Each represents the mean ± 1 SD for the average of 54 leg movements for control and 51 leg movements for RPNI per increment of pressure. Positive trends in both RPNI and Control groups imply RPNI transduced EMG signals of proportional intensity similar to that of an in situ Control

Proportional control of a neuroprosthesis requires the ability to distinguish variations in EMG peak recordings from volitional behavior. Using this tenet, EMG amplitude was mapped 1:1 to the speed of prosthetic hand movement [32]. Increasing von Frey monofilament pressure led to an observable increase in rat hind limb movement intensity. Rats in the Control and RPNI groups had a positive logarithmic correlation between von Frey filament forces (intensity of stimulus), EMG amplitude, and therefore the instantaneous voltage used to actuate the prosthetic hand. This positive correlation enabled a pre-programmed, graded control of the prosthetic hand speed (R^2^ = 0.802, *p* < 0.05 and R^2^ = 0.758, p < 0.05, respectively) (Fig. 4).

**Fig. 5:**
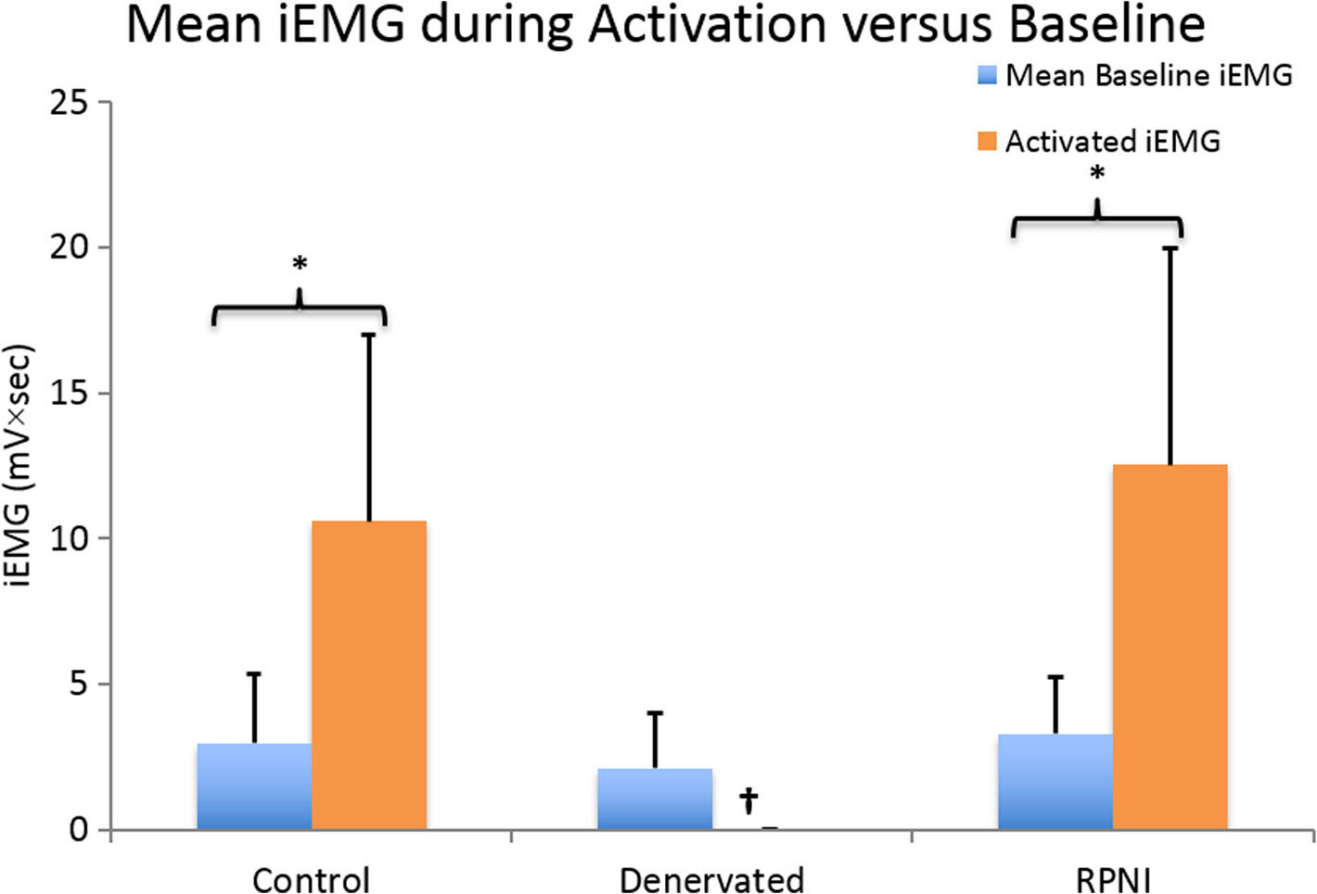
Mean ± 1 Standard Deviation of iEMG values obtained during baseline (blue) and activation trials regardless of monofilament pressure (orange) in Control, Denervated and RPNI rat cohorts. iEMG is calculated as the area under the curve measured during consecutive 300 msec intervals of EMG signal acquisition during testing. Activated iEMG is recorded during rat movement while baseline iEMG is obtained during rest. † Denervated group as expected did not show activity during rat movement; therefore, no activated iEMG was calculated. A * indicates significantly higher activation signals, when compared with relative baseline signals within Control and RPNI groups (*p* < 0.05)

As expected, no significant correlations were found between resting “baseline” iEMG activity and the monofilament pressure subsequently used for either the Control or RPNI groups (R^2^ = 0.12 and R^2^ = 0.19, respectively). This is expected, as changes in “baseline” iEMG activity results from biologic and electronic variation, whereas increased iEMG activity during activation is due to increased muscle activation, contraction, and movement, not random variation (Fig. 5).

## Discussion

Regenerative peripheral nerve interfaces (RPNI) provide a biologic connection to peripheral nerves to amplify efferent motor action potentials producing high-fidelity motor control signals and favorable signal to noise ratios. In this study, we have demonstrated reliable RPNI signal transduction in real-time EMG signals obtained during voluntary muscle activation. To date, this is the first study to demonstrate both real time and proportional control of a myoelectric prosthesis using an RPNI.

The amplitude based direct control algorithm strategy determined for this study was modelled using simple linear regression. While there are many means of quantifying muscle activity using myoelectric signals [33], integrated EMG was chosen as the proportional input to the controller, as it has been shown to be a reliable quantifier of muscle force [34]. In order to reset the integration to zero, a 300 millisecond acquisition window was employed; the window timespan was chosen to ensure that at maximum opening velocity (300 mm/s), the prosthetic hand does not exceed its opening width (100 mm) [35]. To ensure that the prosthetic gripper’s activation does not occur as a result of background noise or previous myoelectric activity, a running threshold was computed using a weighted average of the myoelectric signals recorded during previous acquisition windows. Consequently, the prosthetic hand was actuated only if the integrated EMG signal obtained during the current sampling window was one standard deviation above threshold [25]; during actuation, the gripper’s velocity was proportional to the amount of discrete standard deviations that iEMG lay above threshold.

An important criterion in patient satisfaction resides in the reaction time of the prosthetic device [24, 36–38]. The algorithm in this study integrated EMG signals over 300 millisecond intervals, building an acceptable 300 millisecond delay into the prosthesis activation time. Integrating EMG signals over this period reduced errant prosthetic activation due to random variation in baseline EMG. Future studies utilizing RPNI interfaces with alternative methods for prosthesis activation can reduce this built-in delay. In the present study, prosthetic hand speed was increased with increasing amplitude of iEMG signals. A strong, positive correlation existed between iEMG signal amplitude and the monofilament force applied to the rat’s limb (i.e. the experimental stimulus). This is pivotal, as adjusting both speed and directional movement of a prosthetic device restores greater functionality to the amputee.

One of the primary challenges in neural interfaces is long-term durability in performance. The current study shows that RPNIs were safe and effective in the rat hind limb. As expected, post-experimental gross evaluation of the lateral thigh compartment revealed that the free muscle grafts were healthy in RPNI group rats, but were severely atrophic in the Denervated group. Furthermore, the consistently low EMG signals derived from the Denervated group signify that RPNI EMG activity is not affected by motion artifact or crosstalk from neighboring muscles.

The implantation procedures were well tolerated, consistent with previous RPNI implantation surgeries [27–29, 39]. Within the lifespan of the rat, we have observed minimal to no signal degradation over at least 7 months post implantation [29]. The electrodes implanted in the RPNI in this study were stainless steel, and interfaced directly with transferred skeletal muscle, thereby avoiding direct contact with the peripheral nerve and corresponding biofouling of the electrode, possible neural inflammation and injury. While muscle tissue tolerates the presence of epi- or intra-muscular electrodes fairly well, electrode materials and designs are currently being investigated to continue to minimize the inflammatory response. [40, 41]

The study’s purposes included proof that signals transduced from only one RPNI are suitable to control a one degree of freedom (DOF) myoelectric hand. RPNI technology with multiple implanted RPNIs would also be applicable for prostheses capable of many DOFs. As devised, each RPNI is anatomically “hard-wired” from select motor control areas of the brain through peripheral nerves to individual RPNIs. Consequently, multiple RPNI EMG signals, or co-activation, could be decoded using linear regression control or parallel multi-site control as accomplished with signals available in TMR [42]. Control strategies such as amplitude based direct control, as well as sequential and simultaneous pattern recognition have been studied with able bodied and TMR patients [32]. Those who study efficient control may find that providing several strategies may allow a neuroprosthesis user to achieve fine actuation with direct control, and larger movements with simultaneous control [42].

There are inherent limitations to demonstrating the feasibility of myoelectric prosthesis control using a rat model. Limiting RPNI implantation to one RPNI per one rat hind limb allows for only a simple model which limits prosthetic functionality to single axis actuation. RPNIs are currently being implanted in humans, on multiple individual nerve branches, to provide numerous independent DOF. In this manner, each RPNI will contribute to several movements of a prosthesis when the transduced EMG signals are processed using pattern recognition.

Finally, in this study, we valuated outcomes 135 days after RPNI surgery with electrode implantation. This time-point was selected based on previous studies showing RPNI revascularization, muscle fiber regeneration, and reinnervation occurring at 120 days [29]. Future longitudinal studies of RPNI control of a neuroprosthetic device are currently assessing the lifetime efficacy of RPNI signal transduction.

## Conclusion

This study validated an algorithm for translating EMG signals from RPNIs for real-time control of a neuroprosthetic hand. Signal contamination from muscles adjacent to the RPNI was minimal. The EMG signals were successfully acquired from RPNIs and translated into real-time neuroprosthetic control via an algorithm that allowed for concrete demonstration that RPNIs provide reliable proportional control of the neuroprosthesis. RPNI myoelectric hand control was both sensitive and specific.
